# How to express visually the genetic identity of a scientific journal? – an example of the *Croatian Medical Journal* cover page

**DOI:** 10.3325/cmj.2015.56.179

**Published:** 2015-06

**Authors:** Maša Vukmanović, Srećko Gajović

**Affiliations:** 1 Studio Maša Vukmanović, Zagreb, Croatia; 2 *Croatian Medical Journal*, Zagreb, Croatia

## Visual identity of the *CMJ*

The *Croatian Medical Journal* (*CMJ*) pays a lot of attention to its visual identity. This visual identity includes not only fonts, colors, page organization, logo, but all the published items. The cover page, as the most noticeable part of the *CMJ’s* visual identity, is considered to be a record of the journal’s activities, history, and progress. Every issue of the *CMJ* has a specially designed cover page that corresponds to the main topic of the issue. In this vein, the title page of the current issue is related to the 9th International Society for Applied Biological Sciences (ISABS) Conference to be held in June 2015 at the Croatian island of Brač.

## DNA as a hereditary molecule of the *CMJ*

The topic of the current issue is Forensic and Anthropologic Genetics and Individualized Medicine. In order to design the cover page, our designer (Maša Vukmanović) identified key words – forensics, genetics, and individual, and tried to find the visual analogues for their combination. The *CMJ* was considered as an object of biomedical analysis and in this way was transformed from a scientific journal to a virtual patient. The humanized *CMJ* as a subject of forensic study, in order to reveal its genetic (or visual?) identity was expected to provide its DNA. As the journal does not abruptly change from issue to issue, one can agree that its hereditary information is carried through the time and its generations. This hereditary information in the real word does not correspond to any biological molecule, but it is in the domain of the *CMJ*’s cultural history. Still, for this cover page and this particular topic, it was assumed that the DNA was a hereditary molecule determining the *CMJ*.

## How to analyze *CMJ*’s DNA?

**Figure 1:**
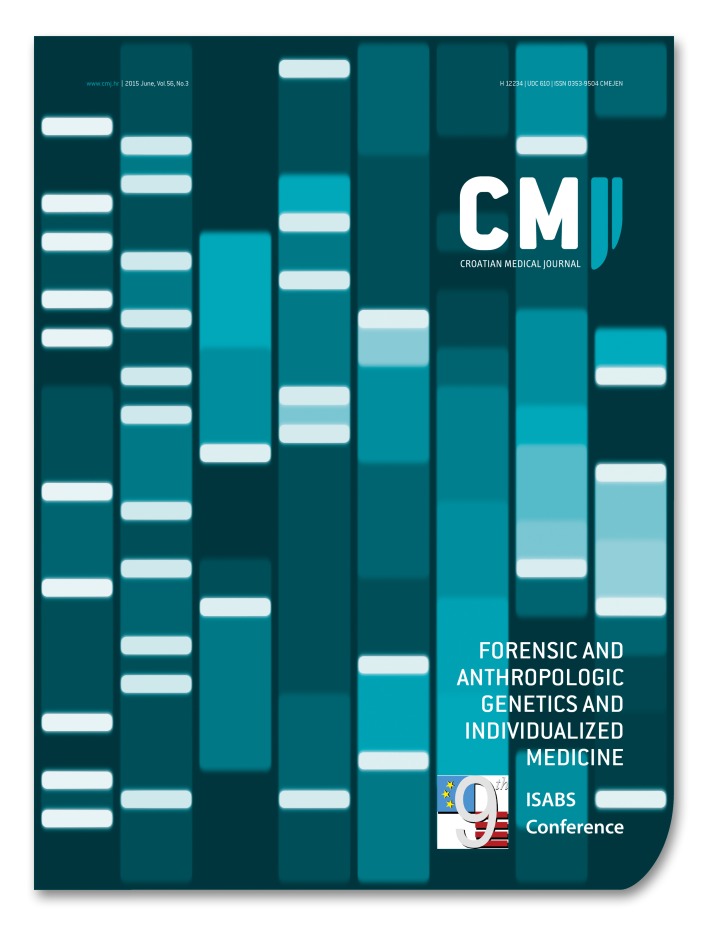
The cover page of *CMJ* issue 56(3).

To assess the *CMJ’s* DNA, the cover page designer decided to look for *CMJ’s* genes represented in the published articles. The structure of the articles was analyzed and every article was transformed into a ladder of signals obtained by electrophoresis (Figure 1, full resolution cover page Supplementary figure 1 (Supplementary figure 1)). The articles were distributed in lanes and different colors were assigned to different parts of the articles. Using this procedure, a visual pattern was obtained representing the current article structure, similar to real life electrophoretic DNA analysis. Moreover, the result of the analysis represents the individuality of the *CMJ*, and at the same time corresponds to its identity, enabling its potential forensic identification.

## Science and arts

The analogies of science and arts are frequently evoked (1). Art can be used as a result of the scientific analysis, or as a tool to achieve research or therapeutic goals (2). In the *CMJ*, we strongly believe that the unity of science and arts can offer substantial benefits not only to our journal, but also to science and human culture in general.
