# Supplementary material for: How to express visually the genetic identity of a scientific journal? – an example of the Croatian Medical Journal cover page
Source: Croat Med J. 2015 Jun;56(3):179–80. doi: 10.3325/cmj.2015.56.179 (PMC4500969; doi:10.3325/cmj.2015.56.179)
Supplement: Supplementary Figure 1 [file CroatMedJ_56_s008.pdf]

www.cmj.hr | 2015 June, Vol.56, No.3

H 12234 | UDC 610 | ISSN 0353-9504 CMEJEN

CMJ

CROATIAN MEDICAL JOURNAL

FORENSIC AND ANTHROPOLOGIC GENETICS AND INDIVIDUALIZED MEDICINE

9th ISABS Conference
